# Investigation of Factors Influencing the Fear of Cancer Recurrence in Breast Cancer Patients Using Structural Equation Modeling: A Cross-Sectional Study

**DOI:** 10.1155/2022/2794408

**Published:** 2022-12-06

**Authors:** Hai-Tao Guo, Shuang-Shuang Wang, Chun-Fang Zhang, Hong-Jie Zhang, Min-Xiang Wei, Yu Wu, Chen-Xiao Su

**Affiliations:** ^1^Department of Operation, Affiliated Hospital of Hebei University, Baoding, China; ^2^Department of Radiation Oncology, Affiliated Hospital of Hebei University, Baoding, China; ^3^Department of Administrative, Affiliated Hospital of Hebei University, Baoding, China; ^4^College of Public Health Hebei University, Baoding, China; ^5^Department of Breast Surgery, Affiliated Hospital of Hebei University, Baoding, China

## Abstract

**Objective:**

This study aimed to investigate the fear of cancer recurrence (FCR) in breast cancer patients and develop a structural equation model of influencing factors to help formulate clinical intervention strategies.

**Methods:**

A convenience sample of 325 patients was surveyed using a general and disease-related data questionnaire, which combined the Fear of Progression Questionnaire-Short Form, Mishel Uncertainty in Illness Scale, Perceived Social Support Scale, and Medical Coping Modes Questionnaire.

**Results:**

The total score of FCR in breast cancer patients was 35.06 ± 10.83, and 53.8% of patients reached the clinical level. The structural equation model demonstrated that illness uncertainty had a direct positive impact on FCR (*β* = 0.275, *p* < 0.05), and it could have an indirect impact through social support and resignation coping methods (*β* = 0.254, *p* < 0.05).

**Conclusion:**

The fear of cancer recurrence in breast cancer patients needs further understanding. Medical staff can reduce or buffer FCR in breast cancer patients by strengthening positive influences, such as social support, or weakening negative influences, such as illness uncertainty and resignation coping.

## 1. Introduction

According to a 2020 report by the American Cancer Society [1], breast cancer is one of the most common malignancies among women, representing a serious threat to their future health. With the advance of medical technology, the treatment of breast cancer is improving although recurrence and metastasis are common problems that breast cancer patients face after diagnosis and treatment [2, 3]. A related study [4] revealed that more than half of breast cancer patients suffer from fear of cancer recurrence (FCR) after their rehabilitation. This refers to a psychological state of fear, worry, or anxiety that cancer may reappear or progress [5]. The occurrence of FCR can seriously affect patient health, reduce the quality of life for patients and their families, cause excessive use of medical resources, and increase the potential costs to the medical system [6]. Disease uncertainty, social support, and coping strategies have been reported as some of the essential influencing factors for FCR in breast cancer patients [7]. Chinese scholars Ye et al. [8] found that disease uncertainty in breast cancer patients was positively correlated with FCR, and good social support was an essential external resource to promote mental health. Other studies [8, 9] have reported that FCR tends to be lower in breast cancer patients with better social support. Another factor influencing FCR is the patient's coping style, which describes how the individual behaves and responds to their health problems; various coping styles may have different effects on patients. A study [10] revealed that breast cancer patients who take more effective coping strategies had lower FCR levels, while those with negative coping styles, such as avoidance, had higher FCR levels. Most of these studies adopted correlation or regression analyses to confirm the effects of these factors on FCR in breast cancer patients. However, the internal mechanisms of influencing factors and FCR remain unclear. As a statistical method, a structural equation model can deal with the relationship between multiple independent and dependent variables and clarify the relationship structure among factors. Therefore, in this study, based on the stress coping theory and literature review, structural equation modeling was applied. This helped to analyze the impact path and effects of disease uncertainty, social support, and coping styles on FCR, which may allow clinical staff to better understand FCR in breast cancer patients and implement appropriate psychological interventions.

## 2. Methods

### 2.1. Design and Procedure

This study adopted a convenience sampling method, enrolling hospitalized breast cancer patients in surgery, chemotherapy, and radiotherapy departments from June to December 2019. Inclusion criteria are as follows: (1) patients aged ≥18 years old; (2) clinical diagnosis of stage I–IV breast cancer; (3) patients who were aware of their condition; and (4) patients who were informed of the study's purpose and provided informed consent to participate voluntarily. Exclusion criteria are as follows: (1) breast cancer had metastasized from other malignancies rather than being the primary cancer; (2) patients with a previous history of mental illness or severe psychological or cognitive impairment; (3) patients with dyslexia relating to reading, writing, and verbal communication; and (4) patients who were critically ill and could not cooperate.

After the review and approval of the Hospital Ethics Committee and the consent of relevant departments, the investigators went to the appropriate departments to carry out their study. Before commencing, the researchers explained the purpose, significance, and methods to the patients who met the research criteria. After obtaining each patient's consent, the consent form was signed, and the investigation was carried out in a quiet and undisturbed environment. The patient filled out the questionnaire anonymously. While filling out the questionnaire, the researchers took time to clarify any topics prone to misunderstanding and answered patients' questions; this helped to ensure the accuracy and authenticity of the data. After completing the questionnaire, the forms were collected on the spot, and the accuracy of the questionnaire was checked at the same time. Questionnaires with regular answers or missing answers greater than 20% were eliminated. If the form was incomplete, it was filled in again, returned after checking, and then numbered. In this study, 340 questionnaire forms were distributed, and 325 valid questionnaires were finally recovered. The validity rate of the questionnaires was 95.6%.

### 2.2. Measures

#### 2.2.1. Research Tools

1. General demographics and disease-related data: Based on the literature review, the researchers selected general demographic, sociological, and related disease variables that might affect the FCR of breast cancer patients. These included age, nationality, religious beliefs, marital status, whether the patient had children, place of residence, occupation, educational level, family's per capita monthly income, payment method of medical expenses, and primary caregivers. The disease-related data included whether or not the patients have had surgery, other treatment methods used, tumor stage, and disease frequency.
2. The Fear of Progression Questionnaire Short Form (FoP-Q-SF): This scale was compiled by Mehnert et al. [11] and is mainly used to evaluate the fear of disease progression in patients with cancer and other chronic diseases. In 2015, Wu et al. [12] translated the scale into Chinese for the first time and completed cultural adjustments to form a simplified scale for fear of disease progression in China. The scale includes 12 items covering aspects of physical health and social/family life and adopts a 5-point Likert scale method (1 for “never” and 5 for “always”), with a total score of 12–60. The higher the score, the higher the fear of disease progression. When the score is ≥34, it means that it has reached the defined level of clinical significance; the patient has psychological dysfunction and requires corresponding intervention measures. The Cronbach's *α* coefficient of this scale is 0.883. In the present study, Cronbach's *α* coefficient was 0.909, the physical health dimension was 0.809, and the social family dimension was 0.882.
3. Mishel's Uncertainty in Illness Scale (MUIS): This scale was initially developed by a nursing scientist Mishel [1] and is widely used abroad. It mainly measures the uncertainty of an illness in adult inpatients. This study adopted the Chinese version of the MUIS developed by Xu and Huang in Taiwan [13]. There are 25 items on the scale, including two dimensions uncertainty and complexity factors. Scores are measured using a 5-point Likert scale: 1 means strongly disagree and 5 means strongly agree; the scale's total score is 25–125 points. The higher the patient's score, the higher the uncertainty surrounding the disease. In the present study, Cronbach's *α* coefficient was 0.919, the uncertainty factor dimension was 0.923, and the complexity dimension was 0.78.
4. The Perceived Social Support Scale (PSSS): This scale was compiled by Zimet et al. [14]. It measures the level of support perceived by the individual from various aspects of society. Its total score reflects the total degree of social support felt by an individual. The total Cronbach's *α* coefficient of this scale was 0.992. The adjusted PSSS developed by Chinese scholars Huang et al. [15] was adopted in this study. The scale includes two dimensions such as in-family support (4 items) and extra-family social support (8 items). The score is calculated using a 7-point Likert scale, where a score of 1 means strongly disagree and 7 means strongly agree. The total score of the scale ranges from 0 to 84; the higher the score, the higher the level of social support. In the present study, the total Cronbach's *α* coefficient of the scale was 0.93, and the in-family support dimension was 0.69, and the extra-family social support dimension was 0.964.
5. Medical Coping Modes Questionnaire: This questionnaire was developed by the American scholar Feifel et al. [16] and is mainly used to evaluate the coping style of patients in the face of disease. In 2000, Shen and Jiang [17] translated the scale into Chinese. The questionnaire includes three dimensions such as acceptance, avoidance, and resignation, covering a total of 20 items. The score is measured using a 4-point Likert scale: the higher the score for a coping method, the more frequently it is adopted. The Cronbach's *α* coefficients of dimensions were 0.69, 0.60, and 0.76, respectively. In the present study, Cronbach's *α* coefficient of the acceptance dimension was 0.724, the avoidance dimension was 0.557, and the resignation dimension was 0.614.

#### 2.2.2. Construction of the Hypothetical Model

**Figure 1:**
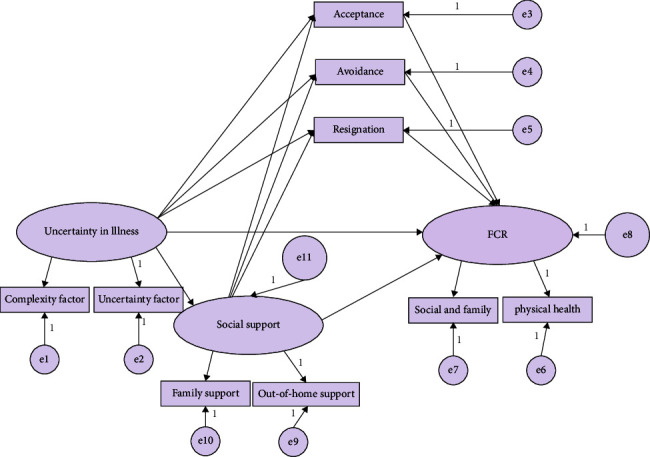
Assumption diagram of structural equation model of influencing factors for FCR in breast cancer patients.

In this study, based on the literature and theoretical analysis, the hypothetical model was formed using the stress coping theory by Lazarus and Folkman; this included two important psychological processes such as cognitive assessment and response. Coping methods include taking positive action, avoiding, letting things go, seeking information and help, and applying psychological defense mechanisms. Coping resources include an individual's functional status, social support system, and individual capabilities. In this model, it is pointed out that the disease uncertainty of breast cancer patients affects their psychosocial adaptation, with social support and coping mode acting as mediating factors; furthermore, social support plays a regulatory role in the patients' coping. The FCR in breast cancer patients was regarded as an endogenous latent variable, and the two dimensions of FCR (physical health and social family) were taken as the observation variables. The uncertainty in illness in breast cancer patients was regarded as an exogenous latent variable; its two dimensions (uncertainty and complexity factors) were regarded as observation variables. Social support and coping style were regarded as external variables, whereas the two dimensions of social support (family and outside-of-family support) were regarded as the observation variables. Using all these variables, the study constructed the assumption diagram of the structural equation model of influencing factors for FCR in breast cancer patients (Figure 1).

#### 2.2.3. Statistical Analysis

Data were inputted by two persons. SPSS 22.0 statistical software and Amos 21.0 structural equation model software were used to analyze the data. Normally distributed measurement data were expressed as mean ± standard deviation (*x* ± SD). Count data were expressed as frequency and percentage. Pearson's correlation analysis was employed to explore the correlation between breast cancer patients' FCR and uncertainty in illness, social support, and coping styles. Finally, Amos 21.0 software was used to fit, analyze, and modify the hypothetical structural equation model. The inspection level was set at *α* = 0.05, with *p* < 0.05 considered as being statistically significant.

## 3. Results

### 3.1. General Demographics and Disease-Related Data

A total of 340 questionnaires were distributed, 325 of which were recovered and 15 of which were disqualified (for regular answers); the response rate of questionnaires was 95.6%. As shown in Table 1, there were significant differences in FoP-Q-SF scores depending on the patient's age, marital status, place of residence, occupation, education level, family income, medical expenses, and tumor stage (*p* < 0.05). However, there were no significant differences relating to ethnicity, religious belief, presence or absence of offspring, major caregivers, surgery, and morbidity (*p* > 0.05). See Table 1 for details.

### 3.2. Breast Cancer Patients' Scores of Disease Uncertainty, Social Support, and Coping Styles

In 325 breast cancer patients, the total score of MUIS was 69.40 ± 14.49, the uncertainty factor dimension was 43.17 ± 10.45, and the complexity dimension was 26.23 ± 5.67. The total score of PSSS was 61.36 ± 16.23, where the score of the family support dimension was 21.67 ± 6.42, and the score of extra-family social support was 39.69 ± 10.99. In the coping style, the score of the acceptance dimension was 19.98 ± 3.38, the avoidance dimension was 17.16 ± 2.60, and the resignation dimension was 9.64 ± 3.61.

### 3.3. The Score of Fear of Cancer Recurrence in Breast Cancer Patients

In breast cancer patients, the total score of FoP-Q-SF was 35.06 ± 10.83 points, the score of the physical health dimension was 18.22 ± 5.65, and the society/family dimension score was 16.84 ± 5.87. The score of the physical health dimension was slightly higher than society/family. There were 175 patients with a total score of ≥34, accounting for 53.8%. See Table 2 for details.

### 3.4. Correlation Analysis between Fear of Cancer Recurrence and Uncertainty in Illness, Social Support, and Coping Style in Breast Cancer Patients

Pearson correlation analysis showed that the total score of FCR was positively correlated with the total score of disease uncertainty (*p* < 0.01, *r* = 0.624). The total score of FCR was negatively correlated with the total score of social support (*p* < 0.01, *r* = −0.29). The total score of fear of recurrence was negatively correlated with acceptance coping (*p* < 0.01, *r* = −0.245) and was statistically significant for resignation coping (*p* < 0.01, *r* = 0.51). See Table 3 for details.

### 3.5. Fitting and Modification of the Structural Equation Model of Fear of Cancer Recurrence Influencing Factors

**Figure 2:**
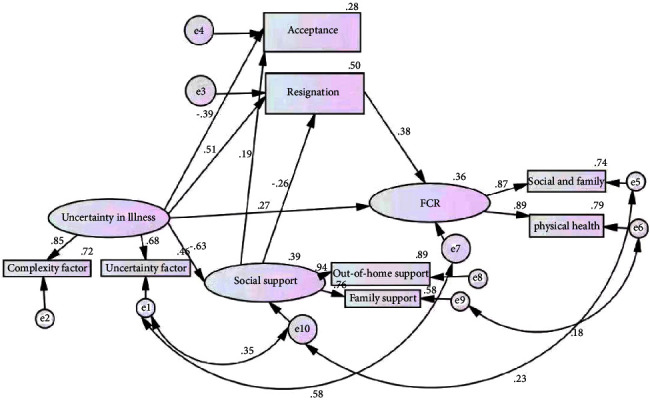
Modified diagram of structural equation model of influencing factors for FCR in breast cancer patients.

Correlation analysis results revealed that the avoidance-based coping style did not influence FCR, so this path in the hypothetical model was removed. Amos 21.0 software was used to fit the hypothetical model with the maximum likelihood method. The paths without statistical significance were removed, and the model was adjusted by modifying the index. Therefore, the structural equation model of influencing factors for FCR in breast cancer patients was obtained (Figure 2). The fitting index of the modified structural equation model is presented in Table 4. The effect of uncertainty in illness, social support, and resignation coping on FCR is shown in Table 5.

### 3.6. Model Fitting and Modification

Amos 21.0 software was used for the initial model fitting. It was assumed that the GFI, NFI, and IFI of the model met the acceptable standards of model adaptation, while the other values did not. The model was modified using the following steps.

1. According to the model fitting results, the path of “social support for fear of recurrence of cancer” (*p* > 0.05) had no statistical significance, so this path was deleted. According to the modified index, if error terms E1 and E10 established a covariant relationship, the chi-square value decreased by 26.989. Therefore, the co-variant was established between them, and after reanalysis, the chi-square value decreased to 77.415. However, at *p* < 0.05, AGFI increased to 0.848 < 0.9, while RMSEA decreased to 0.124. If 0.08 was still not up to standard, it was further revised.
2. According to the modified index, if the covariant relationship was established between error terms E1 and E7, the chi-square value was reduced by 10.539; as such, the covariant value was established. After reanalysis, the chi-square value reduced to 26.665 (*p* < 0.05), which did not meet the adaptation standard of AGFI = 0.943 > 0.9. Meeting the adaptation standard, RMSEA decreased to 0.061 < 0.08, and so further modifications were made.
3. According to the modified index, if the covariant relationship between error terms E5 and E10 was established, the chi-square value was reduced by 5.475; as such, the path was established. After reanalysis, the chi-square value was reduced to 20.509 (*p* < 0.05), which did not meet the adaptation standard. The test results of other indicators were optimized for further modification.
4. According to the modified index, if the covariant relationship between error terms E6 and E9 was established, the chi-square value was reduced by 4.087; as such, the path was established. After reanalysis, the chi-square value was 15.854 (*p*=0.104), which reached the adaptation standard.
5. According to the model fitting results, the path of “facing the fear of cancer recurrence” (*p* > 0.05) had no statistical significance, so this path was deleted and reanalyzed. It was assumed that the model fit results met the testing standards.

The model results showed that the parameters were set reasonably, and there were no outliers. From the model identification table, it was found that the basic fit indicators of the modified structural equation model for FCR in breast cancer patients met the test criteria; this indicated that the results did not violate the model identification rules. In terms of the overall model fit, the absolute, value-added, and parsimonious fit indices all met the acceptance criteria of the model.

## 4. Discussion

### 4.1. Pathways Influencing Fear of Cancer Recurrence in Breast Cancer Patients

The results of the structural equation modeling in this study showed that three pathways influenced FCR in breast cancer patients. Pathway 1: Illness uncertainty ⟶ fear of cancer recurrence; Pathway 2: Illness uncertainty ⟶ resignation ⟶ fear of cancer recurrence; and Pathway 3: Illness uncertainty ⟶ social support ⟶ resignation ⟶ fear of cancer recurrence. Korean researchers Kim and So [18] conducted a study on 198 breast cancer patients and developed a structural equation model, the results of which showed three pathways where disease uncertainty could affect the level of psychosocial adjustment in breast cancer patients. These are Path 1: uncertainty ⟶ psychological adjustment level; Path 2: uncertainty ⟶ social support ⟶ psychological adjustment level; and Path 3: uncertainty ⟶ optimism ⟶ coping ⟶ psychological adjustment level. The present study is similar; in that, it explores the psychological impact of uncertainty on breast cancer patients by developing a structural equation model. However, this study explored how illness uncertainty affects a specific psychological state of fear in breast cancer patients, which differs from psychosocial adjustment and is also more specific in terms of the pathways leading to the psychological state.

### 4.2. Analysis of the Effects of Various Factors Influencing Fear of Cancer Recurrence in Breast Cancer Patients

The level of disease uncertainty in breast cancer patients can directly influence the level of FCR. This study also showed that, in addition to the direct positive effect, uncertainty also had an indirect effect on FCR. In terms of effect values, the direct effect of disease uncertainty on FCR was not significantly different from the indirect effect. It is suggested that when disease uncertainty influences FCR indirectly through other factors, these factors may have a cumulative effect that is comparable to the direct effect of disease uncertainty on FCR. Through a survey of 180 hospitalized breast cancer patients, Ye et al. [19] found that FCR in breast cancer patients was positively correlated with disease uncertainty; this result was the same for the present study. In addition to corroborating these findings, this study also revealed the specific pathways through which disease uncertainty can influence FCR by constructing a structural equation model. Thus, the results can provide theoretical support for clinical staff who wish to reduce FCR and improve the psychological well-being of breast cancer patients.

The results of the structural equation model showed that social support was an indirect influence on FCR, with social support mediated by submission coping having an indirect negative effect on the level of FCR. In a survey of 192 inpatients, Xing et al. [20] found that FCR was negatively associated with social support. Also, in a study of 180 breast cancer patients, Ye et al. [19] found that FCR in breast cancer patients was negatively correlated with social support. This suggests that when patients face the heavy blow of a cancer diagnosis and uncomfortable treatments, good social support is an important mental health resource. The care and assistance of friends and relatives help to relieve patients of adverse emotions during treatment, reduce psychological pressure, improve patients' compliance with treatment, and enhance their confidence in overcoming the disease. Therefore, while providing knowledge about the disease, healthcare professionals should encourage patients to strengthen communication with friends, colleagues, and other patients through different social activities. This may enhance their confidence in the treatment and optimize the role of social support in disease treatment.

Additionally, the results of structural equation modeling showed a direct effect of the submission coping style on FCR, where breast cancer patients who adopted this coping had increased levels of FCR. In their survey study of 228 breast cancer patients, Cai et al. [21] found that submission, or yield, coping was positively correlated with FCR in breast cancer patients, which is reflected in the present study. Adding to this research, the present study sets out a more detailed pathway analysis, which demonstrates how resignation coping may be linked to FCR. The present study has highlighted the importance of breast cancer patients' coping styles. The unpredictability of disease progression and prognosis, and the unknown future of the patient, can lead to compromise and reduced compliance with treatment. It can also cause emotional disturbance, impact the effectiveness of medication, and exacerbate stressful events, resulting in increased levels of FCR. Therefore, clinical and nursing staff should guide breast cancer patients to adopt the correct coping style, thereby reducing FCR and improving patients' quality of life. There are some studies in China which investigated the facing methods like Baduanjin and Qigong Therapy [22, 23].

### 4.3. Limitations

This study only investigated breast cancer inpatients in surgery, radiotherapy, and chemotherapy departments at a tertiary care hospital in Hebei province, which faced problems finding a sufficient representative sample. Additionally, this study used a cross-sectional survey method, which could only determine the factors influencing FCR among breast cancer patients. In the future, multiple methods can be used to develop and validate specific intervention strategies for FCR among breast cancer patients, which may provide more targeted strategies and suggestions for reducing FCR.

## 5. Conclusion

The current situation of FCR in breast cancer patients in China is not optimistic. For more than half of the patients surveyed, FCR reached the clinical level, which suggests the need for clinical intervention. In addition, the structural equation model analysis demonstrated that uncertainty in illness, social support, and resignation were the influencing factors for FCR in breast cancer patients. Uncertainty in illness not only had a direct positive impact on FCR but also had an indirect positive impact through social support and resignation coping; social support had an indirect negative impact on FCR, meaning that patients with higher levels of social support tended to have lower FCR levels; and resignation coping had a direct positive impact on FCR. This suggests that clinical medical staff should pay attention to FCR as it may affect the psychological well-being of breast cancer patients.

## Figures and Tables

**Table 1:** The FoP-Q-SF score of breast cancer patients in general demographic and disease related data $\left(\bar{\chi }\pm s\right)$.

Item	Group	*N* = 325	FoP-Q-SF	*t/F*	*p* value
Age	≤44	96	35.75 ± 10.90	5.166	<0.01^*∗*^
	45 ∼ 59	141	37.21 ± 10.33		
	60 ∼ 74	74	31.14 ± 10.16		
	≥74	14	30.17 ± 13.14		

Nationality	Han	310	34.79 ± 10.66	−1.801	0.073
	Other	15	39.93 ± 13.38		

Religious belief	Yes	303	35.22 ± 10.80	0.986	0.325
	No	22	32.86 ± 11.21		

Marital status	Unmarried	12	42.33 ± 16.91	2.756	0.043^*∗*^
	Married	287	34.49 ± 10.51		
	Divorced	12	38.58 ± 10.30		
	Widowed	14	37.43 ± 9.36		

Presence or absence of offspring	0	19	32.95 ± 17.79	0.787	0.456
	1	151	34.63 ± 10.10		
	>1	155	35.74 ± 10.45		

Place of abode	City	86	33.56 ± 11.83	3.417	0.034^*∗*^
	Town	69	33.28 ± 10.35		
	Rural	170	36.55 ± 10.33		

Occupation	Unemployed	28	34.82 ± 7.97	2.361	0.040^*∗*^
	Farmers	172	36.76 ± 10.58		
	Workers	67	32.70 ± 9.49		
	Individual household	7	34.71 ± 13.71		
	Civil servants and public institutions	32	34.09 ± 14.27		
	Other	19	30.16 ± 11.56		

Education level	Primary and below	73	36.37 ± 9.79	3.039	0.029^*∗*^
	Junior high school	131	36.34 ± 10.37		
	High school and technical secondary school	74	32.04 ± 10.47		
	Junior college or above	47	34.21 ± 13.24		

Family per capita monthly income	<1000 RMB	52	38.38 ± 10.28	3.004	0.031^*∗*^
	1000 ∼ 2999 RMB	165	35.31 ± 10.13		
	3000 ∼ 4999 RMB	79	32.76 ± 11.98		
	≥5000 RMB	29	33.97 ± 11.26		

Payment method of medical expenses	At one's own expense	9	43.79 ± 10.88	5.214	0.002^*∗*^
	Medical insurance for urban residents	27	32.11 ± 11.45		
	Employee medical insurance	93	32.58 ± 11.43		
	New rural cooperative medical insurance	196	36.24 ± 10.09		

Major caregivers	Spouse	183	35.42 ± 10.77	0.526	0.717
	Children	104	34.21 ± 10.21		
	Parents	26	36.92 ± 13.59		
	Hire	1	31.00		
	Other	11	33.09 ± 11.33		

Surgery	No	42	33.88 ± 10.83	-0.757	0.450
	Yes	283	35.24 ± 10.84		

Other treatments	None	72	33.52 ± 11.37	1.168	0.312
	Chemotherapy	249	35.56 ± 10.69		
	Radiation therapy	4	32.00 ± 8.29		

Disease stage	I	52	32.12 ± 11.19	4.002	0.008^*∗*^
	II	126	34.51 ± 9.86		
	III	119	35.65 ± 10.11		
	IV	28	40.54 ± 14.96		

Incidence	First	274	35.03 ± 10.74	−0.111	0.912
	Recurrence	51	35.22 ± 11.41		

Note: ^*∗*^*p* < 0.05.

**Table 2:** Grade of FCR in breast cancer patients and score.

Item	Number of category (n)	Score/constituent ratio (%)	Average of category
Total	12	35.06 ± 10.83	2.92 ± 0.90
Physical health	6	18.22 ± 5.65	3.04 ± 0.94
Society-family	6	16.84 ± 5.87	2.81 ± 0.98

**Table 3:** Correlation analysis between FCR and uncertainty in illness, social support, and coping styles in breast cancer patients.

Item	FCR	Uncertainty in illness	Social support	Acceptance	Avoidance	Resignation
FCR	1					
Uncertainty in illness	0.624^*∗∗*^	1				
Social support	−0.290^*∗∗*^	−0.337^*∗∗*^	1			
Acceptance	−0.245^*∗∗*^	−0.401^*∗∗*^	0.415^*∗∗*^	1		
Avoidance	−0.056	−0.084	0.224^*∗∗*^	0.248^*∗∗*^	1	
Resignation	0.51^*∗∗*^	0.522^*∗∗*^	−0.542^*∗∗*^	−0.357^*∗∗*^	−0.127^*∗*^	1

Note: ^*∗∗*^ for *p* < 0.01, ^*∗*^ for *p* < 0.05.

**Table 4:** Adaptation degree of structural equation model after modification.

Statistical inspection quantity	Fitness reference standard or critical value	Test results	Model fitness determination
Absolute fitness index			
*X* ^2^ value	Significance probability value *p* > 0.05	15.885 *p* > 0.05	Y
Goodness of fit index	>0.90	0.988	Y
Goodness of fit index after adjustments	>0.90	0.961	Y
Root mean square residual and square root	<0.05	1.485	N
Standardized root mean square residual and square root		0.0254	Y
Root mean square error of approximation and square root	<0.05 (good fitness), <0.08 (reasonable fitness)	0.037	Y

Value added fitness index			
Standard fitness index	>0.90	0.988	Y
Nonstandard fitting index	>0.90	0.990	Y
Value added fitness index	>0.90	0.996	Y
Simple fitness index			
Critical number of samples (CN value)	>200	402	Y
NC value (*X*^2^ degree of freedom ratio)	1 < NC < 3 indicates that the model has simple fitness and NC > 5 indicates that the model needs modification	1.444	Y

**Table 5:** Analysis of effect of FCR influencing factors in breast cancer patients.

Independent variable	Dependent variable	Direct effect (*β*)	Indirect effect (*β*)	Total effect (*β*)	95% CI
Uncertainty in illness	FCR	0.275	0.254	0.529	0.412∼0.635
	Social support	−0.628	—	−0.628	−0.718∼−0.535
	Acceptance	−0.389	−0.12	−0.508	−0.604∼−0.395
	Resignation	0.513	0.163	0.676	0.588∼0.754

Social support	FCR	—	−0.098	−0.098	−0.162∼−0.048
	Acceptance	0.19	—	0.19	0.042∼0.35
	Resignation	−0.26	—	−0.26	−0.368∼−0.132

Resignation	FCR	0.375	—	0.375	0.25∼0.499

Note: The coefficients was the intermediate effect value.

## Data Availability

The datasets used and/or analysed during the current study available from the corresponding author on reasonable request.

## Abbreviations

FCR: Fear of cancer recurrence
FoP–Q–SF: Fear of progression questionnaire-short form
MUIS: Mishel' s uncertainty in illness scale
PSSS: Perceived social support scale.
